# 749. Interactive Technologies for Preventing HIV in Title X Clinics in Metropolitan Atlanta

**DOI:** 10.1093/ofid/ofad500.810

**Published:** 2023-11-27

**Authors:** Amalia Aldredge, Deja Er, Katherine M Anderson, Ilse Campos, Veronica Joseph, Melissa Kottke, Peyton Williams, Sara Sullivan, Michael W Brooks, Jessica Sales, Anandi N Sheth

**Affiliations:** Emory University, Atlanta, GA; Emory University School of Medicine, Atlanta, Georgia; Emory University Rollins School of Public Health, Atlanta, Georgia; Emory University Rollins School of Public Health, Atlanta, Georgia; Emory University, Atlanta, GA; Emory University School of Medicine, Atlanta, Georgia; The Family Health Centers of Georgia, Inc., Georgia Family Planning System, Atlanta, Georgia; The Family Health Centers of Georgia, Inc., Georgia Family Planning System, Atlanta, Georgia; The Family Health Centers of Georgia, Inc., Georgia Family Planning System, Atlanta, Georgia; Emory University, Rollins School of Public Health, Atlanta, Georgia; Emory University School of Medicine, Atlanta, Georgia

## Abstract

**Background:**

HIV prevention in women is imperative to end the HIV epidemic in the US. However, pre-exposure prophylaxis (PrEP) uptake in this population has been low, and innovative and feasible approaches are needed. The Title X Family Planning Program provides federal funding for sexual health services and HIV prevention to low income or uninsured individuals. We sought to increase PrEP awareness and reduce barriers to providing PrEP for women in Atlanta. We designed, implemented, and evaluated educational tools (trainings, resources, and technical assistance) for staff at Title X clinics on topics relevant for PrEP care for cisgender women.

Figure 1.

**Figure:**
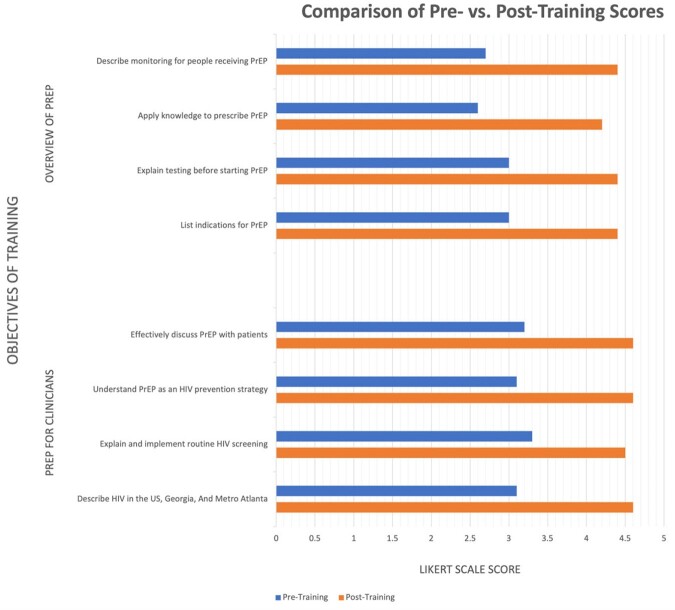

Comparison of pre- and post-training 5-point Likert scale evaluations of ability to meet training objectives for the Overview of PrEP and PrEP for Clinicians Trainings. Two-sample Satterthwaite t-tests comparing pre- and post-training scores were significantly different by individual objective, for each training, and overall (all p<0.001).

**Methods:**

We offered a menu of nine 30-60 minute in-person or virtual trainings (Overview of PrEP, PrEP care for clinicians, PrEP insurance navigation, long-acting PrEP, alternative PrEP delivery models, reporting PrEP metrics, taking a sexual history, STI management, and substance use screening) to staff from 29 Atlanta Title X clinics. We also offered technical assistance with consultation on PrEP implementation planning or any training topic, and patient- and provider-facing resources about PrEP tailored for cisgender women. We used two-sample Satterthwaite t-tests to compare pre- and post- training self-reported ability to meet training objectives by 5-point Likert scale.

**Results:**

In the first year, we provided 15 trainings covering 3 topics to 279 individuals in 20 clinics. We delivered 7 “Overview of PrEP” trainings; participants were medical assistants (31%), nurses (12%), advance practice providers (APPs, 6%), and other (37%). We delivered 7 “PrEP Care for Clinicians” trainings; participants were APPs (20%), physicians (17%), nurses (15%) and other (37%). Staff had significantly higher Likert scores (Figure 1) for ability to meet training objectives after trainings, both overall and by individual objective (all p < 0.001).

**Conclusion:**

We provided guideline-based PrEP trainings tailored for cisgender women to a staff with diverse clinic roles from a wide-reaching network of Atlanta Title X clinics. Ability to meet training objectives substantially increased post-training. Next steps include evaluating program impact on HIV testing, PrEP counseling, and PrEP prescription among cisgender women in Atlanta.

**Disclosures:**

**Melissa Kottke, MD, MPH, MBA**, HRA Pharma: Advisor/Consultant|Organon: Grant/Research Support

